# On Infirmaries and Dispensaries

**Published:** 1829-04-01

**Authors:** 


### INFIRMARIES AND DISPENSARIES.

The ostensible, the paramount object of these institutions is, no doubt, the cure or relief of diseases among the poor. But a secondary and very influential object, is the acquirement and increase of medical knowledge through that best of all channels — EXPERIENCE. Now, we hold it unquestionable that this last object should be promoted as much as possible, and especially where it does not trench on, or interfere with, the primary intention—relief of the pauper. These institutions are supported by the purses of the more opulent classes of the community—and surely these classes have a right to all advantages flowing directly or indirectly from infirmaries and dispensaries, through the medium of improvement in medical science generally. This improvement should not, then, be confined exclusively to those medical officers who actually officiate in such institutions —it should be rendered available by all regularly established practitioners in the vicinity, who may choose to witness the phenomena or the treatment of diseases in public practice. We have received several letters on this subject, and we have been often solicited to draw the attention of the public to it. The following extract of a letter published in the Glasgow FREE PRESS, by Mr. Gibson, of Dumfries, will explain what we allude to in these observations.

"Every one who knows any thing of the medical profession will readily admit, that every surgical operation, almost, is a dissection of the part operated on; and all must confess that one dissection on the living body, when absolutely demanded, is more valuable to the practising surgeon than half a dozen ruch dissections on the dead subject. It is also very obvious, that the inspecting the body of a person who has died of an obscure disease, by a few medical practitioners, after having read over the history of the case, with its daily symptoms and treatment, must prove, in many instances, more advantageous to the community at large, than a dozen dissections in the rooms of the anatomist. The method I would point out is, to permit the *regular medical practitioners* of every town in which there is an Infirmary, or similar establishments, *to have free access to that Infirmary at visiting hours, and full liberty to be present at all operations and dissections performed therein.* Could such a desirable event as the throwing open of the *Infirmaries, the only medical schools in provincial towns*, to respectable practitioners, be accomplished, the most important advantages could not fail to accrue to the medical profession and the public. The rich as well as the poor are subject to disease, and the more enlightened the medical gentlemen of any place are, the more valuable will be their services to those amongst whom they are situated. This praiseworthy system has been acted upon for some time in Glasgow, and the result has been all that could be desired. It has also, I understand, been lately adopted by some of the English Infirmaries. But it is a fact—and a fact, too, unworthy of the age in which we live—that some provincial Infirmaries have positive rules *forbidding* the admission of any medical practitioner, not belonging to the establishment, to be present at operations and dissections, unless *invited* by the surgeons; and I could even point out Infirmaries where the greatest secrecy seems to be used when any operation or dissection is to take place, and the practitioners in the town hear not a word about it, unless, perhaps, they do so some time afterwards by accident. This is any thing but as it should be. If the surgeons of Infirmaries are unable, or afraid, to act before their brethren, they are unfit for the situations which they hold. It has been argued by some of the advocates for the close system, that if medical practitioners were freely admitted, as I propose, they would now and then offend by making remarks. This argument is a libel on the profession. Were any practitioner to conduct himself in an improper or ungentlemanly manner, he would assuredly be expelled by the Committee of Management, which would have the effect of making others more guarded. The practice of *excluding* respectable practitioners from witnessing the treatment, *operations*, and *dissections in the Infirmaries* of the towns in which they reside, at a time when medical science is making such rapid strides, and when a scarcity of subjects to meet the demands of the persevering surgeon has given rise to a series of the most horrible murders that ever stained the annals of a nation, is not only unjust and illiberal, but may even be termed *barbarous*. These establishments, erected and supported at the public expense, were never destined to be made subservient to the purposes of two or three individuals. They were never intended as easy-chairs, for those medical gentlemen who value not, or at least neglect the advancement of their profession, and the diffusion of practical knowledge amongst their brethren. In Glasgow, the surgeons of the Infirmary cannot remain in office longer than two years at one time, nor are they again eligible until after a certain number of years. This is an admirable regulation, and its good effects must be felt alike by surgeon and citizen. During the last year, attempts were made in an English and Scottish infirmary to overthrow the absurd and obnoxious close system, and replace it by the more liberal one so happily pursued in Glasgow; but unfortunately for both the professsion and the public, those concerned found interest enough to have the hole and corner system sanctioned for another year at least. But the Schoolmaster, we are told, is abroad, and it is to be hoped that in his intellectual march through Britain, he will take the trouble to look in upon our different infirmaries, and hold a little discourse with their governors and trustees. I at one time thought of addressing Mr. Secretary Peel on this important subject, and if it is not taken up by some more able person, I may possibly still do so before the meeting of Parliament."

Although we agree with Mr. Gibson in the greater number of sentiments contained in the foregoing extract, we cannot forbear making a remark or two *en passant*.

We are convinced that it would be absurd—at all events, entirely useless, to apply to Mr. Peel on such a subject as the regulation of provincial infirmaries and dispensaries. That gentleman has other and more important matters on hand. Besides, the government can claim no possible control over the internal arrangements of a charity supported by private subscription, and to which no part of the public money goes. All salutary regulations, therefore, must originate with the parties themselves. The practitioners of towns in which these institutions are formed should become annual governors, and, consequently, supporters of them. They could then claim a *right* of visiting them when they pleased, the same as the governors of metropolitan hospitals. We believe the temporary appointments of medical officers would be more advantageous to the public at large, and certainly to the profession, than permanent appointments—and we cannot but lament the niggardly and illiberal policy which could lead to laws, *prohibiting* neighbouring practitioners from visiting infirmaries, or being present at operations! It is a sad indication of the state of feeling in the medical profession! But can we wonder at such precautions, when we see open marts for anonymous calumny? A visitor at one of these institutions, who may entertain a grudge against his neighbour, may blast his character with perfect security to himself. He has only to dispatch an anonymous charge against the professional skill of his contemporary, or even his colleague, and it will find prompt insertion in the "JOURNAL of DEFAMATION." Mr. Gibson may say this is a "libel on the profession." It is so—but the libel is, unfortunately, TRUE. It is occurring every day. We trust, however, that, as a better feeling obtains in medical society, the cause of these prohibitory laws, or, as Mr. G. rather coarsely terms them, the "hole and corner system," will be withdrawn, and that every institution, of the kind we allude to, will be thrown open to all respectable medical practitioners in their vicinity.

---

